# Recognition of MI-EEG signals using extended-LSR-based inductive transfer learning

**DOI:** 10.3389/fninf.2025.1559335

**Published:** 2025-04-09

**Authors:** Zhibin Jiang, Keli Hu, Jia Qu, Zekang Bian, Donghua Yu, Jie Zhou

**Affiliations:** ^1^Department of Computer Science and Engineering, Shaoxing University, Shaoxing, China; ^2^Institute of Artificial Intelligence, Shaoxing University, Shaoxing, China; ^3^Information Technology R&D Innovation Center of Peking University, Shaoxing, China; ^4^Department of Computer Science and Artificial Intelligence, Changzhou University, Changzhou, China; ^5^Department of AI & Computer Science, Jiangnan University, Wuxi, China; ^6^Department of Taihu Jiangsu Key Construction Lab of IoT Application Technologies, Wuxi, China

**Keywords:** motor imagery, EEG, brain-computer interface, LSR, inductive transfer learning

## Abstract

**Introduction:**

Motor imagery electroencephalographic (MI-EEG) signal recognition is used in various brain–computer interface (BCI) systems. In most existing BCI systems, this identification relies on classification algorithms. However, generally, a large amount of subject-specific labeled training data is required to reliably calibrate the classification algorithm for each new subject. To address this challenge, an effective strategy is to integrate transfer learning into the construction of intelligent models, allowing knowledge to be transferred from the source domain to enhance the performance of models trained in the target domain. Although transfer learning has been implemented in EEG signal recognition, many existing methods are designed specifically for certain intelligent models, limiting their application and generalization.

**Methods:**

To broaden application and generalization, an extended-LSR-based inductive transfer learning method is proposed to facilitate transfer learning across various classical intelligent models, including neural networks, Takagi-SugenoKang (TSK) fuzzy systems, and kernel methods.

**Results and discussion:**

The proposed method not only promotes the transfer of valuable knowledge from the source domain to improve learning performance in the target domain when target domain training data are insufficient but also enhances application and generalization by incorporating multiple classic base models. The experimental results demonstrate the effectiveness of the proposed method in MI-EEG signal recognition.

## 1 Introduction

A brain–computer interface (BCI) is a technology that establishes connections between the brain and external devices, facilitating information exchange between them (Edelman et al., 2024). BCIs collect and analyze electrical signals generated by brain activity, transforming these signals into instructions that can be used to control external devices such as computers, prosthetics, and wheelchairs. As such, BCIs can assist, enhance, and repair human sensory and motor functions, improving human–computer interaction capabilities. BCIs do not rely on the peripheral nervous system or muscles, providing a new method for people who have lost their mobility due to illness or disability to communicate with the external environment and operate devices. BCIs not only open new possibilities for people with disabilities but also advance our understanding of the brain, ushering in a new era of human–computer interaction.

### 1.1 Motivation

Motor imagery electroencephalographic (MI-EEG) (Mohammadi et al., 2022) signal recognition is an important mechanism for brain-computer interfaces (BCIs). Moreover, with the advancement of machine learning, numerous classification methods based on machine learning have been proposed for MI-EEG signal recognition in the literature (Abbas et al., 2021; Ko et al., 2021; Zhang et al., 2024; Ghumman et al., 2021; Cover and Hart, 1967; Aldea et al., 2014; Kohavi, 1996; Wang and Zhang, 2016; Fisher, 1936; Li et al., 2022; Bennett and Demiriz, 1999; Fouad et al., 2020; Siddiqa et al., 2024; Siddiqa et al., 2023; Qureshi et al., 2022; Qureshi et al., 2023), including neural networks (NNs) (Abbas et al., 2021; Ko et al., 2021), fuzzy logic systems (FLSs) (Zhang et al., 2024; Ghumman et al., 2021), k-nearest neighbors (kNNs) (Cover and Hart, 1967; Aldea et al., 2014), naïve Bayes (NB) (Kohavi, 1996; Wang and Zhang, 2016), linear discriminant analysis (LDA) (Fisher, 1936; Li et al., 2022), support vector machines (SVMs) (Bennett and Demiriz, 1999; Fouad et al., 2020), and more. Although these methods have demonstrated varying degrees of success, they typically require a large amount of subject-specific training data to adjust their parameters. However, this data acquisition process can be time-consuming and not user-friendly. When calibration data is insufficient, the classification performance of these algorithms can significantly deteriorate. As highlighted in BCI Competition III (Blankertz et al., 2006), “*a challenge is that more expectations of training a model with a good classification accuracy are becoming urgent in the case that only a small number of training samples are available.*” Therefore, it is essential to develop advanced machine-learning methods for MI-EEG that perform effectively with small calibration datasets.

**Figure 1:**
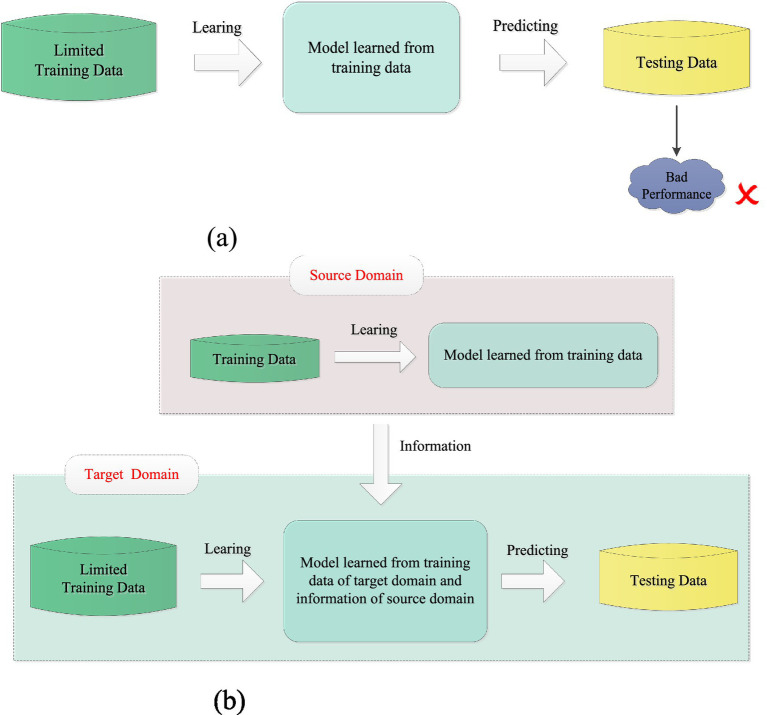
Differences between traditional machine learning **(a)** and transfer learning **(b)**.

Transfer learning is a promising method for addressing the above problem. It can be used to transfer useful information from related scenes (i.e., source domains) to the current scene (i.e., target domain), which typically has limited training data (Pan and Yang, 2010). As a result, transfer learning is particularly effective in improving classification performance during the early stages of model training when there is not enough subject-specific training data. Figure 1 shows the differences between traditional machine learning and transfer learning. Since its introduction in 1995, transfer learning has been successfully applied in classification, clustering, and regression, with classification being the most extensively researched area. Some representative studies can be found in Zhang et al. (2022), Jiang et al. (2019), Xie et al. (2018), Pan et al. (2011), Wan et al. (2021), Li et al., 2019). Existing transfer learning methods can be categorized into three types: inductive transfer learning methods (Zhang et al., 2022; Jiang et al., 2019), which consider both supervised source and target domains; transductive transfer learning methods (Xie et al., 2018; Pan et al., 2011), which involve supervised source domains and unsupervised target domains; and unsupervised transfer learning methods, which account for both unsupervised source and target domains (Wan et al., 2021; Li et al., 2019). In MI-EEG signal recognition, when labeled MI-EEG samples in the target domain are insufficient, inductive transfer learning methods naturally become the preferred choice. Furthermore, since MI-EEG signals involve personal privacy information, inspired by Jiang et al. (2019), we investigate a knowledge-based inductive transfer learning method to ensure security without directly utilizing samples from the source domain.

**Figure 2:**
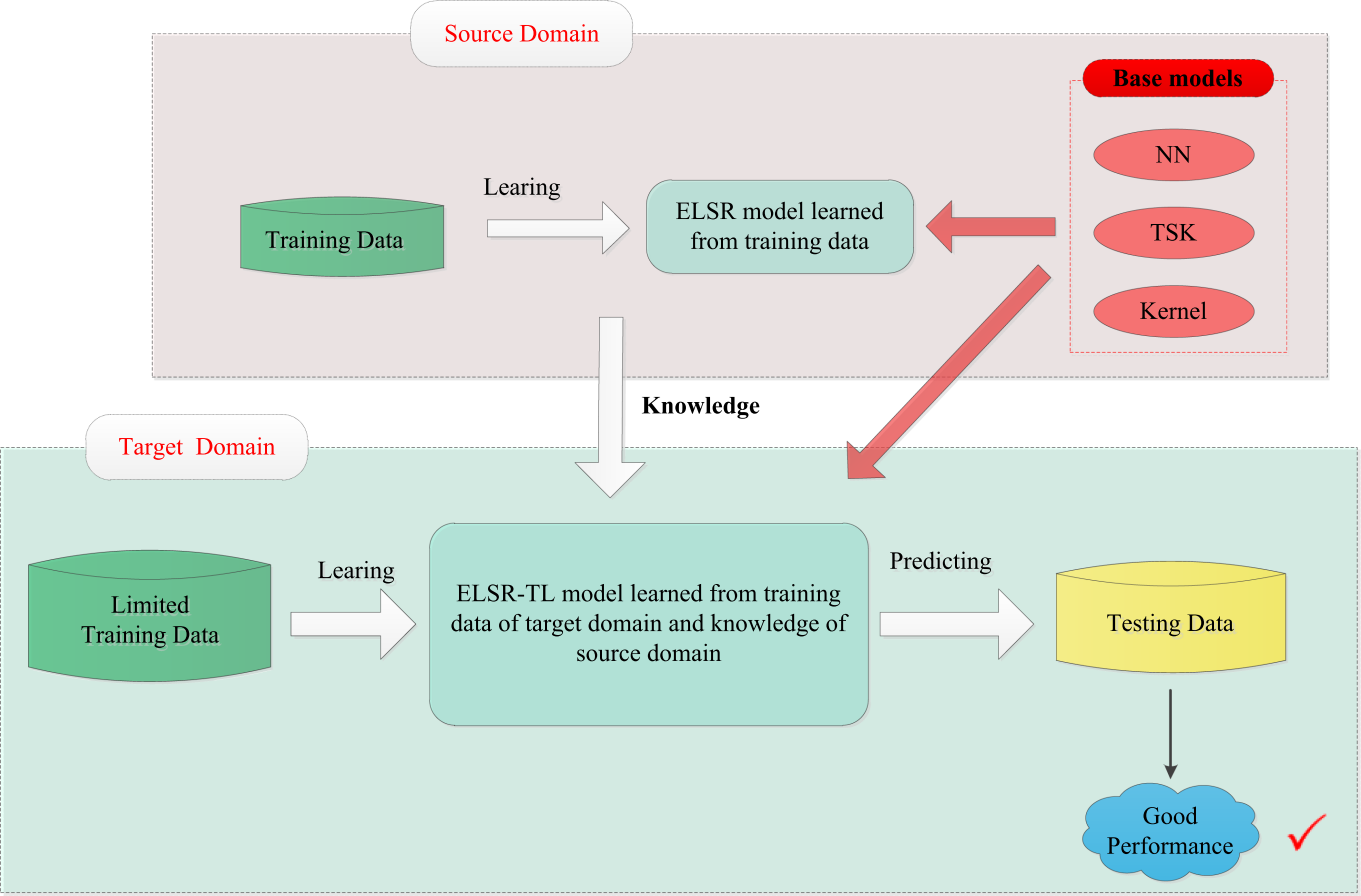
Framework of ELSR-TL.

Inductive transfer learning has recently attracted widespread attention and demonstrated strong performance in MI-EEG signal recognition. However, most existing inductive transfer learning methods are tailored to specific base models, rendering them inapplicable to other base models. As a result, they demonstrate poor performance in terms of application and generalization. To address this limitation, we propose an extended-LSR-based inductive transfer learning framework (ELSR-TL) that integrates neural networks, Takagi-Sugeno-Kang (TSK) fuzzy systems, and kernel methods. Figure 2 shows the framework of ELSR-TL.

### 1.2 Contributions

The main contributions of this study can be highlighted as follows:

1. ELSR-TL has an inductive transfer learning mechanism that can be used to transfer useful knowledge from the source domain to enhance learning performance in the target domain when the training data in the target domain are insufficient.
2. ELSR-TL enhances LSR by integrating multiple classic base models, such as neural networks, TSK fuzzy systems, and kernel methods. As such, ELSR-TL is not only suited for a specific model but also demonstrates improved applicability and generalization.
3. Experimental studies were conducted to validate the applicability of the proposed method for MI-EEG signal identification.

The remainder of this paper is organized as follows: Section II describes related work, including studies on existing MI-EEG feature extraction and pattern recognition methods. Section III details the proposed extended-LSR-based inductive transfer learning method. Section IV provides the experimental results and analysis. Finally, Section VI presents the conclusions drawn.

## 2 Backgrounds

This section states the backgrounds underlying the proposed MI-EEG recognition method. It describes the datasets used to evaluate the method and reviews several classical feature extraction and pattern recognition methods.

### 2.1 Datasets

We used BCI Competition Data Set IVa, provided by Fraunhofer FIRST and Charité University Medicine Berlin. A detailed description of this dataset can be found in (Blankertz et al., 2006).

**Figure 3:**
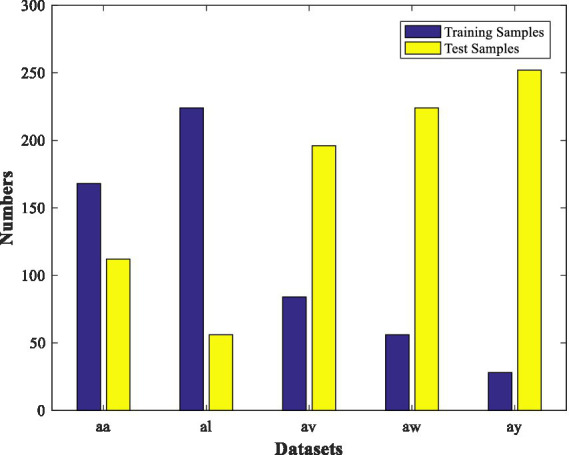
Distribution of each subset from the BCI Competition Data Set IVa.

**Figure 4:**
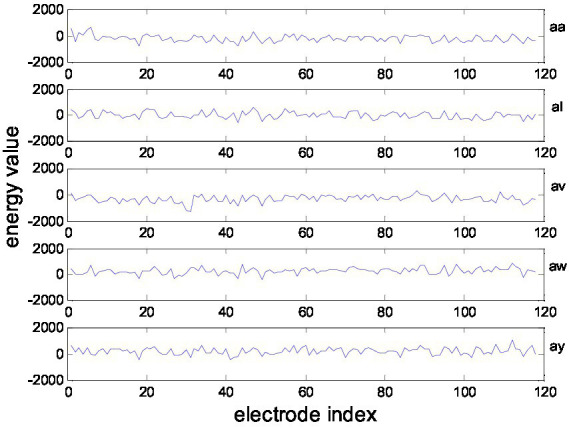
Representative MI-EEG signals for each subset of the BCI Competition Data Set IVa.

This MI-EEG dataset contains five subsets corresponding to five healthy testers (aa, al, av., aw, and ay). Each subset contains 280 EEG trials, which have 128 electrodes and a trial length of 3.5 s. Each subset was partitioned into a training set and a test set, as shown in Figure 3. Figure 4 shows the representative MI-EEG signals in the five subsets.

### 2.2 Feature extraction methods

EEG signals are complex, nonlinear, and non-stationary. Effective feature extraction is critical to pattern recognition performance. Some of the most representative feature extraction methods have been proposed to manage raw MI-EEG signals. Typically, feature extraction methods can be classified into four main categories: time-domain analysis, frequency-domain analysis, time-frequency analysis, and space-domain analysis.

In time domain analysis, EEG signal features are analyzed in the time domain. Characteristics of the waveforms, such as mean, variance, amplitude, and kurtosis, can be used to extract features of MI-EEG signals (Greene et al., 2008).

In frequency domain analysis, the features of EEG signals are analyzed by investigating the relationship between their frequency and energy. The short-time Fourier transform (Schafer and Rabiner, 1973) is a classical power spectrum analysis method, and adaptive autoregression (Pfurtscheller et al., 1998) is an improved frequency domain analysis method.

In time-frequency analysis (Blanco et al., 1997), the features of EEG signals are extracted using the joint distribution information of the time and frequency domains. Wavelet transform analysis (Antonini et al., 1992) is the most representative method in this category.

In space-domain analysis, the features of EEG signals are extracted by analyzing the electrical activity of neurons in different brain spaces. Common spatial pattern (CSP) (Lotte and Guan, 2011) is a commonly used method in this category. In this method, labeled trials are used to produce a transformation that maximizes the variance of one class while minimizing the variance of the other.

### 2.3 Pattern recognition methods

Pattern recognition utilizes the extracted EEG features for classification. Some of the most representative pattern recognition methods include the following: (1) NNs (Abbas et al., 2021; Ko et al., 2021), which simulate the mechanism of the human nervous system. Feedforward NNs are the most commonly used in EEG classification. (2) FLSs (Zhang et al., 2024; Ghumman et al., 2021), which emulate the human reasoning process and excel at managing numerical and linguistic uncertainties. (3) kNNs (Cover and Hart, 1967; Aldea et al., 2014), which determine the class of a new sample by considering its k nearest neighbors. (4) NB (Kohavi, 1996; Wang and Zhang, 2016), a simple and efficient classification algorithm based on probability. By utilizing known conditional probability and *a priori* probability, NB calculates the posterior probability of each class and assigns the test sample to the class with the highest a posteriori probability. (5) LDA (Fisher, 1936; Li et al., 2022), which applies the Fisher criterion to find the optimal projective vector that maximizes the largest scatter between classes while minimizing the scatter within each class. (6) SVMs (Bennett and Demiriz, 1999; Fouad et al., 2020), which aim to maximize the margins between different classes.

Although existing MI-EEG classification methods have demonstrated their effectiveness in various applications, they all require a substantial amount of subject-specific training data. In practice, such training data may not be easy to obtain, and the classification accuracy of existing methods may drop significantly. To address this challenge, we use an inductive transfer learning-based MI-EEG classification method.

## 3 Extended-LSR-based inductive transfer learning

In this section, we provide a detailed description of the proposed extended-LSR-based inductive transfer learning (ELSR-TL) method. First, we extend LSR (Naseem et al., 2010) to its extended version, ELSR, by merging neural networks, TSK fuzzy systems, and kernel methods. Then, we develop the proposed ELSR-TL. Finally, we present the learning algorithm and theoretical analysis of ELSR-TL.

### 3.1 ELSR

#### 3.1.1 Objective function of ELSR

ELSR is an extension of the basic LSR (Naseem et al., 2010). Given *n d*-dimensional samples ${\left\{\left(x_{i},y_{i}\right)\right\}}_{i=1}^{N}$ , where $x_{i}\in {\mathbb{R}}^{d}$ , $y_{i}\in \left\{-1;+1\right\}$ , the objective function of ELSR can be expressed as follows:

(1) $$\min_{w}\frac{1}{2}{\left\|X_{\rho }w-y\right\|}^{2}+\frac{\lambda }{2}{\left\|w\right\|}^{2}$$

where the matrix $X_{\rho }={\left[\rho \left(x_{1}\right),\cdots ,\rho \left(x_{N}\right)\right]}^{T}\in {\mathbb{R}}^{N\times d_{\rho }}$ denotes all the given training samples, and $\rho \left(x\right)\in {\mathbb{R}}^{d_{\rho }}$ is the hidden mapping function in the hidden mapping space. **w** represents the mapping matrix, λ is the given regularization parameter, and **y** is the corresponding label matrix.

The decision-making function of ELSR can be expressed as follows:

(2) $$y=f\left(x\right)=\rho {\left(x\right)}^{T}w$$

Using different mapping functions $\rho \left(x\right)$ , we can integrate multiple models, such as neural networks, TSK fuzzy systems, and kernel methods, into the proposed ELSR framework. In other words, ELSR can be developed for different base models, which improves its generalization and adaptability. We will describe its relationships with several base models next.

##### 3.1.1.1 The relationship between ELSR and feedforward NNs

A multiple hidden layer feedforward network (MHFN) has an input layer, *M* hidden layers, and an output layer. The multiple hidden layers can be treated as a single complex hidden layer, allowing the overall activation function of these hidden layers to be represented by a single complex function. Therefore, an MHFN can be viewed as a generalized single hidden layer feedforward network (SHFN) with a more complex activation function. The output of a generalized SHFN can be expressed as follows:

(3) $$y=f\left(x\right)=\overset{N_{M}}{\underset{i=1}{\sum }}g_{i}{\left(x,\theta_{i}\right)}^{T}w_{i}$$

where $N_{M}$ is the number of nodes in the last hidden layer of an MHFN. As demonstrated in Huang et al. (2006), if the activation function $g_{i}\left(x,\theta_{i}\right)$ is piecewise continuous, then the hidden nodes can be randomly generated independently of the training data, and the corresponding NN still maintains its universal approximation capability. Let the hidden mapping function $\rho \left(x\right)$ as Equation 4:

(4) $$\rho \left(x\right)={\left[g_{1}\left(x,\theta_{1}\right),\cdots ,g_{N_{M}}\left(x,\theta_{N_{M}}\right)\right]}^{T}$$

Then, Equation 3 can be expressed as follows:

(5) $$y=f\left(x\right)=\rho {\left(x\right)}^{T}w$$

Comparing Equation 5 with Equation 2, we can see that Equation 5 is a special case of Equation 2, so Equation 1 can be used to optimize the corresponding MHFN.

##### 3.1.1.2 The relationship between ELSR and TSK fuzzy systems

The Takagi–Sugeno–Kang fuzzy system (Gu et al., 2024; Bian et al., 2024) is the most widely used FLS due to its simplicity and flexibility. The rules in a TSK fuzzy system are typically represented as Equation 6:

TSK Fuzzy Rule $R^{k}$ :

(6) $$\begin{array}{l} \mathrm{IF}\;x_{1}\;\mathrm{is}\;A_{1}^{k}\wedge x_{2}\;\mathrm{is}\;A_{2}^{k}\wedge \cdots \wedge x_{d}\;\mathrm{is}\;A_{d}^{k}\\ \mathrm{Then}\;f^{k}\left(x\right)=p_{0}^{k}+p_{1}^{k}x_{1}+\cdots +p_{d}^{k}x_{d}\;k=1,\cdots ,K \end{array}$$

Here, $A_{i}^{k}$ is a fuzzy set for the *i*th input variable in the *k*th rule, *K* is the number of fuzzy rules, and ∧ is a fuzzy conjunction operator. The output of the TSK fuzzy system is computed as Equation 7:

(7) $$y=f\left(x\right)=\overset{K}{\underset{k=1}{\sum }}\frac{\mu^{k}\left(x\right)}{\overset{K}{\underset{k^{\prime }=1}{\sum }}\mu^{k^{\prime }}\left(x\right)}\cdot f^{k}\left(x\right)=\overset{K}{\underset{k=1}{\sum }}{\tilde{\mu }}^{k}\left(x\right)\cdot f^{k}\left(x\right)$$

where $\mu^{k}\left(x\right)$ is the firing level of Rule $R^{k}$ , and ${\tilde{\mu }}^{k}\left(x\right)$ is the normalized $\mu^{k}\left(x\right)$ , i.e., Equation 8:

(8a) $$\mu^{k}\left(x\right)=\overset{d}{\underset{i=1}{\prod }}\mu_{A_{i}^{k}}\left(x_{i}\right)$$

(8b) $${\tilde{\mu }}^{k}\left(x\right)=\mu^{k}\left(x\right)/\overset{K}{\underset{k^{\prime }=1}{\sum }}\mu^{k^{\prime }}\left(x\right)$$

The parameters of the antecedent fuzzy sets are usually derived from clustering. The output of the TSK fuzzy system can subsequently be expressed as as Equations 9, 10:

(9) $$y=f\left(x\right)=\rho {\left(x\right)}^{T}w$$

where

(10a) $$\rho \left(x\right)={\left[{\left({\tilde{x}}^{1}\right)}^{T},{\left({\tilde{x}}^{2}\right)}^{T},\cdots ,{\left({\tilde{x}}^{K}\right)}^{T}\right]}^{T}$$

(10b) $${\tilde{x}}^{k}={\tilde{\mu }}^{k}\left(x\right)x_{e}$$

(10c) $$x_{e}={\left(1,x^{T}\right)}^{T}$$

(10d) $$w={\left[{\left(p^{1}\right)}^{T},{\left(p^{2}\right)}^{T},\cdots ,{\left(p^{K}\right)}^{T}\right]}^{T}$$

(10e) $$p^{k}={\left(p_{0}^{k},p_{1}^{k},\cdots ,p_{d}^{k}\right)}^{T}$$

Equation 9 suggests that training the TSK fuzzy system can also be treated as a special case of ELSR, and thus, it can be addressed using Equation 1.

##### 3.1.1.3 The relationship between ELSR and kernel methods

A kernel linear regression model is expressed as follows:

(11) $$y=\rho {\left(x\right)}^{T}w$$

The hidden mapping $\rho \left(x\right)$ can be viewed as a kernel function; thus, Equation 11 can also be solved using Equation 1. In this case, ELSR also corresponds to the classical kernel ridge regression (Saunders et al., 1998).

#### 3.1.2 Solution of ELSR

Depending on the condition of the hidden mapping, the objective function of ELSR in Equation 1 can be efficiently solved in various ways $\rho \left(x\right)$ . Here, we discuss the different cases as follows:

*Case 1:* $\rho \left(x\right)$ *is known*: In this case, we can obtain explicit values of the data $\rho \left(x\right)$ in the hidden mapping space.

Let $J\left(w\right)=\min_{w}\frac{1}{2}{\left\|X_{\rho }w-y\right\|}^{2}+\frac{\lambda }{2}{\left\|w\right\|}^{2}$ ; according to the optimization theory (Qu et al., 2023a; Qu et al., 2023b), the solution for the model parameter w can then be obtained by taking the derivatives of Equation 1 and equating them to zero. That is,

(12) $$\begin{array}{l} \frac{\partial J\left(w\right)}{\partial w}=0\\ \Rightarrow X_{\rho }^{T}X_{\rho }w-X_{\rho }^{T}y+\lambda w=0\\ \Rightarrow w={\left(X_{\rho }^{T}X_{\rho }+\lambda I_{d_{\rho }}\right)}^{-1}X_{\rho }^{T}y \end{array}$$

The final decision function $f\left(x\right)$ can then be expressed as Equation 13:

(13) $$y=f\left(x\right)=\rho {\left(x\right)}^{T}w$$

with **w** obtained in Equation 12.

*Case 2:* $\rho \left(x\right)$ *is unknown:* In this case, the explicit formulation of the data $\rho \left(x\right)$ in the hidden mapping space cannot be obtained, meaning that **w** cannot be specified explicitly. Therefore, the kernel trick is necessary to determine the final decision function $f\left(x\right)$ . Although introducing the kernel trick into the solution strategy in Equation 12 is challenging, Equation 14, identity can be adopted to address this issue:

(14) $${\left(P^{-1}+Q^{T}U^{-1}Q\right)}^{-1}Q^{T}U^{-1}=PQ^{T}{\left(QPQ^{T}+U\right)}^{-1}$$

In Equation 14, **P**, **Q**, and **U** are three matrices. Let $P=\frac{1}{\lambda }I_{d_{\rho }}$ , $Q=X_{\rho }$ , and $U=I_{N}$ . With the identity of Equation 14, the solution in Equation 12 can then be expressed as follows:

(15) $$w={\left(X_{\rho }^{T}X_{\rho }+\lambda I_{d_{\rho }}\right)}^{-1}X_{\rho }^{T}y=X_{\rho }^{T}{\left(X_{\rho }X_{\rho }^{T}+\lambda I_{N}\right)}^{-1}y$$

Define a Mercer kernel matrix as Equation 16:

(16) $$\Omega =X_{\rho }X_{\rho }^{T}\in {\mathbb{R}}^{N\times N}\text{,}$$

where $\Omega_{i,j}=\rho {\left(x_{i}\right)}^{T}\rho \left(x_{j}\right)=K\left(x_{i},x_{j}\right)$ , and $K\left(\cdot \right)$ is a kernel function.

The final decision function $f\left(x\right)$ can then be expressed as follows:

(17) $$\begin{array}{l} y=f\left(x\right)=\rho {\left(x\right)}^{T}w=\rho {\left(x\right)}^{T}X_{\rho }^{T}{\left(X_{\rho }X_{\rho }^{T}+\lambda I_{N}\right)}^{-1}\\ y=\left[\begin{array}{l} K\left(x,x_{1}\right)\\ \vdots \\ K\left(x,x_{N}\right) \end{array}\right]{\left(\Omega +\lambda I_{N}\right)}^{-1}y \end{array}$$

### 3.2 ELSR-TL

#### 3.2.1 Objective function of ELSR-TL

ELSR-TL integrates transfer learning and ELSR. Its objective function can be expressed as follows:

(18) $$\min_{w_{t}}\frac{1}{2}{\left\|X_{\rho ,t}w_{t}-y\right\|}^{2}+\frac{\lambda }{2}{{\left\|w\right\|}_{t}}^{2}+\frac{\beta }{2}{\left\|w_{t}-w_{s}\right\|}^{2}$$

where $X_{\rho ,t}\in {\mathbb{R}}^{N_{t}\times d_{\rho }}$ represents $N_{t}$ training samples of $d_{\rho }$ dimensions in the target domain. $w_{t}$ and $w_{s}$ represent the mapping matrices of the target domain and source domain, respectively. λ and β are the given regularization parameters, and **y** is the corresponding label matrix of the target domain.

In Equation 18, the first two terms are inherited directly from ELSR for learning from the target domain data, while the third term is used to leverage knowledge from the source domain. In other words, ELSR-TL generalizes ELSR from the perspective of transfer learning.

Moreover, as a regularization parameter, β can be used to adjust the role of transfer learning. When β is large, it indicates that transfer learning has a significant impact, indicating that the knowledge obtained from the source domain has a significant positive effect on the target domain. In contrast, when β is very small, it indicates that its role in learning of the target domain is relatively small. In extreme cases, when β=0 , it means that β has no effect on the learning of the target domain. In other words, we can control the effectiveness of transfer learning by making adjustments, thus effectively avoiding negative transfer.

#### 3.2.2 Solution of ELSR-TL

ELSR-TL is solved differently in different scenarios:

*Case 1:* $\rho \left(x\right)$ *is known*: In this case, we can obtain explicit values of the data $\rho \left(x\right)$ in the hidden mapping space. The solution for the model parameter $w_{t}$ can then be obtained in a similar form as that shown in Equation 12, that is

(19) $$\begin{array}{l} \frac{\partial J\left(w_{t}\right)}{\partial w_{t}}=0\\ \Rightarrow X_{\rho ,t}^{T}X_{\rho ,t}w_{t}-X_{\rho ,t}^{T}y+\lambda w_{t}+\beta \left(w_{t}-w_{s}\right)=0\\ \Rightarrow w_{t}={\left(X_{\rho ,t}^{T}X_{\rho ,t}+\left(\lambda +\beta \right)I_{d_{\rho }}\right)}^{-1}\left(X_{\rho ,t}^{T}y+\beta w_{s}\right) \end{array}$$

The final output of the proposed ELSR-TL is expressed as follows:

(20) $$y=f\left(x\right)=\rho {\left(x\right)}^{T}w_{t}$$

with $w_{t}$ obtained in Equation 19.

*Case 2:* $\rho \left(x\right)$ *is unknown:* In this case, the explicit formulation of the data $\rho \left(x\right)$ in the hidden mapping space cannot be obtained, and thus, $w_{t}$ cannot be specified explicitly. Similar to the form shown in Equation 17, the output of the proposed ELSR-TL can be calculated using the kernel trick. From Equation 15, we know that $w_{s}$ can be expressed as Equation 21:

(21) $$w_{s}=X_{\rho ,s}^{T}{\left(X_{\rho ,s}X_{\rho ,s}^{T}+\lambda_{s}I_{N_{s}}\right)}^{-1}y_{s}$$

Here, $w_{s}$ is the parameter of ELSR in the source domain. For a similar scenario, let

(22) $$\alpha_{s}={\left(X_{\rho ,s}X_{\rho ,s}^{T}+\lambda_{s}I_{N_{s}}\right)}^{-1}y_{s}$$

A Mercer kernel matrix is defined, and Equation 22 can then be re-expressed as Equation 23:

(23) $$\alpha_{s}={\left(\Omega_{s}+\lambda_{s}I_{N_{s}}\right)}^{-1}y_{s}$$

where $\Omega_{s}=X_{\rho ,s}X_{\rho ,s}^{T}={\left[K\left(x_{i,s},x_{j,s}\right)\right]}_{N_{s}\times N_{s}}$ , in which $K\left(x_{i,s},x_{j,s}\right)$ is the kernel function.

$w_{s}$ can then be written as follows:

(24) $$w_{s}=X_{\rho ,s}^{T}\alpha_{s}$$

From Equation 19, we obtain the following:

(25) $$\begin{array}{c} w_{t}=\frac{\beta }{\lambda +\beta }w_{s}\\ +\frac{1}{\lambda +\beta }X_{\rho ,t}^{T}{\left(X_{\rho ,t}X_{\rho ,t}^{T}+\left(\lambda +\beta \right)I_{N_{t}}\right)}^{-1}\left(y-\frac{\beta }{\lambda +\beta }X_{\rho ,t}w_{s}\right) \end{array}$$

Substituting Equation 24 into Equation 25 and defining a Mercer kernel matrix, the equation above can then be rewritten as follows:

(26) $$\begin{array}{c} w_{t}=\frac{\beta }{\lambda +\beta }w_{s}+\frac{1}{\lambda +\beta }X_{\rho ,t}^{T}\\ {\left(X_{\rho ,t}X_{\rho ,t}^{T}+\left(\lambda +\beta \right)I_{N_{t}}\right)}^{-1}\left(y-\frac{\beta }{\lambda +\beta }X_{\rho ,t}w_{s}\right)\\ \;=\frac{\beta }{\lambda +\beta }X_{\rho ,\;s}^{T}\alpha_{s}+\frac{1}{\lambda +\beta }X_{\rho ,t}^{T}\\ {\left(\Omega_{t}+\left(\lambda +\beta \right)I_{N_{t}}\right)}^{-1}\left(y-\frac{\beta }{\lambda +\beta }\Omega_{t,s}\alpha_{s}\right)\\ \; \end{array}$$

where $\Omega_{t}=X_{\rho ,t}X_{\rho ,t}^{T}={\left[K\left(x_{i,t},x_{j,t}\right)\right]}_{N_{t}\times N_{t}}$ , $\Omega_{t,s}=X_{\rho ,t}X_{\rho ,s}^{T}={\left[K\left(x_{i,t},x_{j,s}\right)\right]}_{N_{t}\times N_{s}}$

Finally, by using $w_{t}$ obtained in Equation 26, the decision function of the proposed ELSR-TL can be expressed as follows:

(27) $$\begin{array}{c} f\left(x\right)=\rho {\left(x\right)}^{T}w_{t}\\ \;=\rho {\left(x\right)}^{T}\left[\frac{\beta }{\lambda +\beta }X_{\rho ,\;s}^{T}\alpha_{s}+\frac{1}{\lambda +\beta }X_{\rho ,t}^{T}{\left(\Omega_{t}+\left(\lambda +\beta \right)I_{N_{t}}\right)}^{-1}\left(y-\frac{\beta }{\lambda +\beta }\Omega_{t,s}\alpha_{s}\right)\right]\\ \;=\frac{\beta }{\lambda +\beta }{\left[\begin{array}{l} K\left(x_{1,s},x\right)\\ \vdots \\ K\left(x_{N_{s},s},x\right) \end{array}\right]}^{T}\alpha_{s}+\frac{1}{\lambda +\beta }{\left[\begin{array}{l} K\left(x_{1,t},x\right)\\ \vdots \\ K\left(x_{N_{t},t},x\right) \end{array}\right]}^{T}{\left(\Omega_{t}+\left(\lambda +\beta \right)I_{N_{t}}\right)}^{-1}\left(y-\frac{\beta }{\lambda +\beta }\Omega_{t,s}\alpha_{s}\right) \end{array}$$

### 3.3 Learning algorithm of ELSR-TL

Considering the above discussion, we summarize the learning algorithm of ELSR-TL in Algorithm 1. Below, we provide some remarks on ELSR-TL.

**Algorithm 1:**
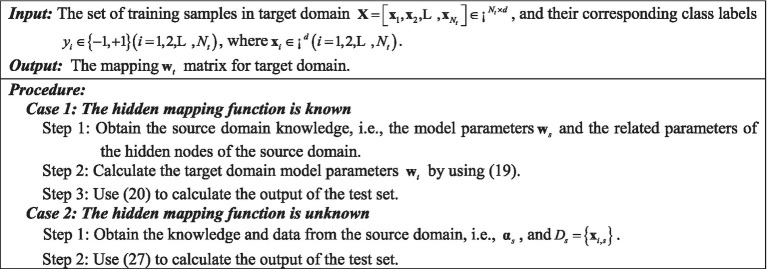
The ELSR-TL.

#### 3.3.1 Remark 1

For the proposed ELSR-TL, if the hidden mapping is known and the amount of training data exceeds the dimensionality of the hidden mapping features (i.e., ( $N_{t}\gg d_{\rho ,t}$ ), obtaining the solution using Equation 25 is more efficient than that using Equation 19, due to the computational complexity of matrix; otherwise, Equation 19 is more efficient.

#### 3.3.2 Remark 2

When the hidden mapping is known, only the knowledge $w_{s}$ is used for transfer learning, and the data in the source domain are not required. This means that the proposed method provides good privacy protection. However, if the hidden feature mapping is unknown, the data in the source are also required, as shown in Equations 26, 27, to effectively implement transfer learning. In this case, the proposed method can no longer protect the privacy of the data in the source domain.

#### 3.3.3 Computational Complexity

In this section, we discussed the computational complexity of Algorithm 1 as follows:

When the hidden mapping is known, the complexity of computing step 1 is about $O\left(d_{\rho ,s}^{3}+d_{\rho ,s}^{2}N_{s}\right)$ , where $d_{\rho ,s}$ is the dimension of samples and $N_{s}$ is the number of samples in the source domain. The complexity of computing the target domain model parameters $w_{t}$ in step 2 is about $O\left(d_{\rho ,t}^{3}+d_{\rho ,t}^{2}N_{t}\right)$ , where $d_{\rho ,t}$ is the dimension of samples and $N_{t}$ is the number of samples in the target domain. In this case, the computational complexity of Algorithm 1 is about $O\left(d_{\rho ,s}^{3}+d_{\rho ,s}^{2}N_{s}+d_{\rho ,t}^{3}+d_{\rho ,t}^{2}N_{t}\right)$ . When the hidden mapping is unknown, the computational complexity of Algorithm 1 is about $O\left(N_{s}^{3}+N_{t}^{3}\right)$ .

## 4 Experiments

In this section, we adopted a real MI-EEG dataset to evaluate the performance of the proposed ELSR-TL method. Moreover, we compared it with seven non-transfer learning methods—LSR (Naseem et al., 2010), KNN (Cover and Hart, 1967), SVM (Bennett and Demiriz, 1999), NB (Kohavi, 1996), CNN (Zhang et al., 2019), ELSR (NN), ELSR (TSK), and ELSR (Ker)—alongside two transfer learning methods—Au-SVM (Wu and Dietterich, 2004) and Tr-Adaboost (Dai et al., 2007). The comparison was conducted in terms of both average classification accuracy and standard deviation for 10 runs. The details of the experimental settings and the MI-EEG recognition results are provided as follows.

### 4.1 Data preparation and feature extraction

#### 4.1.1 Configurations of source and target domains

To match the transfer learning task, we constructed 20 different transfer learning datasets by subject-to-subject transferring. Table 1 shows the 20 different configurations of source and target domains. All source domains have the same number of training data, but the target domains do not. Please note that in our experiments, non-transfer learning methods are only used on the target domain.

**Table 1:** Settings of the source domain and target domain.

Source domain		Target domain		
Datasets	Size	Datasets	Size	
			Training	Test
al	280	aa	168	112
av	280	aa		
aw	280	aa		
ay	280	aa		
aa	280	al	224	56
av	280	al		
aw	280	al		
ay	280	al		
aa	280	av	86	196
al	280	av		
aw	280	av		
ay	280	av		
aa	280	aw	56	224
al	280	aw		
av	280	aw		
ay	280	aw		
aa	280	ay	28	252
al	280	ay		
av	280	ay		
aw	280	ay		

#### 4.1.2 Feature extraction

As mentioned in the background section, effective feature extraction is critical to pattern recognition performance. Based on (Lotte and Guan, 2011), we primarily used the Tikhonov regularization-based common spatial pattern (TR-CSP) (Lotte and Guan, 2011) for feature extraction. Furthermore, we conducted simple experiments using two other feature extraction methods, namely Composite CSP (C-CSP) and Filter Bank CSP (FB-CSP), to compare with TR-CSP. The three feature extraction methods are briefly introduced as follows:

1. TR-CSP: It introduces a quadratic regularization into the CSP objective function and replaces the feature matrix of the new data with the prior knowledge matrix. This regularization prefers filters with smaller norms, reducing the influence of noise.
2. C-CSP: It aims to perform subject-to-subject transfer by regularizing the covariance matrices using data from other subjects. Within the framework of this study, it relies only on the β hyperparameter and defines the generic covariance matrices according to the covariance matrices of other subjects.

FB-CSP: This is a feature extraction method used for motor imagery classification in BCI. It improves the accuracy of motion imagery classification by combining CSP and filter bank techniques, optimizing the subject-specific frequency band for CSP.

**Figure 5:**
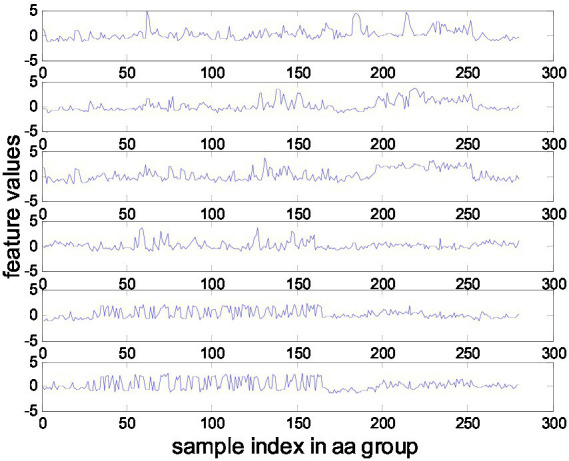
Features extracted from subset aa by TR-CSP.

We extracted features from the time segment between 0.5 and 2.5 s after the cue instructing the subject to perform MI. Each trial is bandpass filtered in the 8–30 Hz range using a fifth-order Butterworth filter. For TR-CSP, we applied three pairs of filters, as recommended in (Lotte and Guan, 2011). Some examples of features extracted from subset aa are shown in Figure 5.

### 4.2 Adopted methods and parameters settings

All the adopted methods (without CNN) are listed in Table 2. Based on the guidelines in (Jiang et al., 2019; Xie et al., 2018; Zhang X. et al., 2023) and our experiments, we employ a grid search strategy to identify the appropriate parameters for all the adopted methods. Table 2 also includes a list of the grid search ranges for each parameter related to all the adopted methods.

**Table 2:** The parameter setting of different methods.

Methods	Parameter settings for grid search
LSR (Naseem et al., 2010): Learns a linear regression model by using each class of training samples with the ${𝓁}_{2}$ -norm regularization.	The regularization parameter $\lambda \in \left\{10^{-6},10^{-5},\ldots ,10^{5},10^{6}\right\}$
kNN (Cover and Hart, 1967): A classical supervised learning model and has been widely used for classification and regression analysis.	The number of nearest points: $k\in \left\{1,3,5,7,9\right\}$ .
SVM (Bennett and Demiriz, 1999): A classical classification method based on kernel trick and margin maximization.	The tradeoff parameter $C\in \left\{2^{-6},2^{-5},\cdots ,2^{0},2^{1},\cdots ,2^{5},2^{6}\right\}$ ; the width in the Gaussian kernel function $\sigma \in \left\{2^{-6},2^{-5},\cdots ,2^{0},2^{1},\cdots ,2^{5},2^{6}\right\}$
NB (Kohavi, 1996): A classification method based on Bayes’ theorem and independent assumption of feature conditions.	The tradeoff parameter $\alpha \in \left\{10^{-5},10^{-4},10^{-3},10^{-2},10^{-1},10^{0}\right\}$
ELSR (NN): Applying the proposed ELSR for signal layer neural networks.	The number of hidden nodes $P\in \left\{10,20,30,40,50,75,100,150,200\right\}$ , the parameters of the sigmoid function: $\kappa \in \left\{2^{-6},2^{-5},\cdots ,2^{0},2^{1},\cdots ,2^{5},2^{6}\right\}$ , and the regularization parameters: $\lambda \in \left\{10^{-6},10^{-5},\ldots ,10^{5},10^{6}\right\}$
ELSR (TSK): Applying the proposed ELSR for TSK.	The number of fuzzy rules: $M\in \left\{5,10,15,20,25,30,40,50,80,100\right\}$ ; the regularization parameter: $\tau \in \left\{10^{-6},10^{-5},\ldots ,10^{5},10^{6}\right\}$ , the regularization parameters: $\lambda \in \left\{10^{-6},10^{-5},\ldots ,10^{5},10^{6}\right\}$ .
ELSR (Ker): Applying the proposed ELSR for the kernel method.	The width in the Gaussian kernel function $\sigma \in \left\{2^{-6},2^{-5},\cdots ,2^{0},2^{1},\cdots ,2^{5},2^{6}\right\}$ and the regularization parameters: $\lambda \in \left\{10^{-6},10^{-5},\ldots ,10^{5},10^{6}\right\}$ .
Au-SVM (Wu and Dietterich, 2004): an inductive transfer learning method based on the linear programming support vector machine with the RBF-type kernel function by using the auxiliary data.	The tradeoff parameter $C\in \left\{2^{-6},2^{-5},\cdots ,2^{0},2^{1},\cdots ,2^{5},2^{6}\right\}$ ; the width in RBF kernel function $\sigma \in \left\{2^{-6},2^{-5},\cdots ,2^{0},2^{1},\cdots ,2^{5},2^{6}\right\}$
Tr-Adaboost (Dai et al., 2007): an inductive transfer learning method based on the LS-SVM learner with the RBF-type kernel function for classification.	The tradeoff parameter $C\in \left\{2^{-6},2^{-5},\cdots ,2^{0},2^{1},\cdots ,2^{5},2^{6}\right\}$ ; the width in RBF kernel function $\sigma \in \left\{2^{-6},2^{-5},\cdots ,2^{0},2^{1},\cdots ,2^{5},2^{6}\right\}$
ELSR-TL (NN): Applying the proposed method for transfer learning of signal layer neural networks.	The number of the hidden nodes: $P\in \left\{10,20,30,40,50,75,100,150,200\right\}$ , the parameters of sigmoid function: $\kappa \in \left\{2^{-6},2^{-5},\cdots ,2^{0},2^{1},\cdots ,2^{5},2^{6}\right\}$ , the regularization parameters: $\lambda \in \left\{10^{-6},10^{-5},\ldots ,10^{5},10^{6}\right\}$ , $\lambda \in \left\{10^{-6},10^{-5},\ldots ,10^{5},10^{6}\right\}$ .
ELSR-TL (TSK): Applying the proposed method for transfer learning of TSK.	The number of fuzzy rules: $M\in \left\{5,10,15,20,25,30,40,50,80,100\right\}$ ; the regularization parameters: $\tau \in \left\{10^{-6},10^{-5},\ldots ,10^{5},10^{6}\right\}$ , $\lambda \in \left\{10^{-6},10^{-5},\ldots ,10^{5},10^{6}\right\}$ , $\lambda \in \left\{10^{-6},10^{-5},\ldots ,10^{5},10^{6}\right\}$ .
ELSR-TL (Ker): Applying the proposed method for transfer learning of the kernel method.	The width in the Gaussian kernel function $\sigma \in \left\{2^{-6},2^{-5},\cdots ,2^{0},2^{1},\cdots ,2^{5},2^{6}\right\}$ , the regularization parameters: $\lambda \in \left\{10^{-6},10^{-5},\ldots ,10^{5},10^{6}\right\}$ , $\lambda \in \left\{10^{-6},10^{-5},\ldots ,10^{5},10^{6}\right\}$ .

### 4.3 Performance indices

The classification accuracy defined Equation 28 is used to evaluate the performances of different methods:

(28) $$\mathrm{Accuracy}=\frac{\left(TP+TN\right)}{\left(TP+TN+FP+FN\right)}$$

### 4.4 Results and discussions

**Figure 6:**
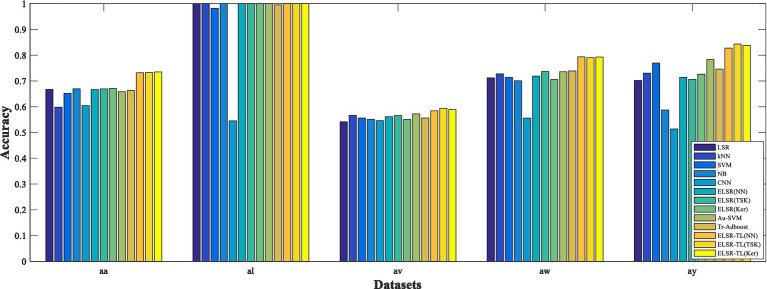
Classification accuracies of 13 different methods, where the accuracies of the transfer learning methods represent the average accuracies across four different source domains with a fixed target domain.

In all the experiments, each comparison method is implemented for 10 runs to report the average classification performance. The experimental results are shown in Tables 3, 4 and Figure 6. Please note that the feature extraction method used for these results is TR-CSP. We can make the following observations:

- In general, the performance of the proposed ELSR-TL-based methods significantly surpasses that of the other methods used, whether they are non-transfer learning methods such as LSR, kNN, SVM, NB, CNN, and ELSR-based methods, or transfer learning methods such as Au-SVM and Tr-Adaboost. This provides experimental evidence that ELSR-TL effectively enhances MI-EEG recognition through knowledge transfer from the source domain to the target domain.
- Comparing the performances of the seven non-transfer learning methods, we can see that the performance of ELSR (TSK) is the best, while the performance of NB is inferior. Moreover, each method obtains significant performance differences on different datasets. Specifically, seven non-transfer learning methods obtain the best performance on dataset *al* but poor performance on datasets *aw* and *ay*. This is because these methods require a large amount of training samples to achieve satisfactory performance, while their performance decreases when there are few training samples.
- Table 4 shows the performances of five transfer learning methods, showing that ELSR-TL (TSK) performs the best while Tr-Adaboost performs the worst. Furthermore, each method achieves similar performances for each target subject, regardless of the auxiliary subject chosen as the source domain. Additionally, for a fixed configuration of the source and target domains, the three ELSR-TL-based methods yield similar classification accuracies.
- Comparing the performances of transfer learning methods (i.e., Au-SVM and ELSR-TL-based methods) with their corresponding non-transfer learning methods (i.e., SVM and ELSR-based methods), we can see that the transfer learning methods outperform the others. Therefore, transfer learning strategies are effective for MI-EEG signal recognition. Impressively, even the least effective transfer learning methods still perform better than or are comparable to the non-transfer learning methods.
- When datasets *aa*, *al*, and *av* are used in the target domain, the performance improvements achieved by the ELSR-TL-based methods are not very significant. This is because the target domain contains sufficient data to train a good model; therefore, the knowledge from the source domain is not critical. However, when datasets *aw* and *ay* are used in the target domain, the ELSR-TL-based methods significantly outperform the other methods due to the limited training data available in the target domain.
- To visually compare the performances of all methods, Figure 6 illustrates the performance of each method across all datasets for visual comparison. Please note that, for transfer learning methods, we report the average performance of four different source domains with fixed target domains in Figure 6. For each dataset, the ELSR-TL-based methods achieve either the best accuracy or performance comparable to that of the other methods.

**Table 3:** Classification accuracies of the non-transfer learning method.

**Datasets**	LSR	kNN	SVM	NB	CNN	ELSR (NN)	ELSR (TSK)	ELSR (Ker)
aa	0.6673 (0.0133)	0.5982 (0.0148)	0.6518 **(0.0071)**	0.6696 (0.0101)	0.6041 (0.0136)	0.6664 (0.0106)	0.6693 (0.0110)	**0.6708** (0.0107)
al	**1(0)**	**1(0)**	0.9821 (0.0031)	**1(0)**	0.5450 (0.0029)	**1(0)**	**1(0)**	**1(0)**
av	0.5416 (0.0115)	**0.5663** **(**0.0132)	0.5561 (**0.0064)**	0.5510 (0.0124)	0.5459 (0.0028)	0.5612 (0.0127)	0.5658 (0.0122)	0.5508 (0.0103)
aw	0.7122 (0.0139)	0.7277 (0.0129)	0.7143 (0.0158)	0.7009 (0.0125)	0.5556 (0.0072)	0.7188 (**0.0103**)	**0.7366** (0.0109)	0.7054 (0.0128)
ay	0.7019 (0.0142)	0.7302 (0.0172)	**0.7698** **(**0.0145)	0.5873 (0.0129)	0.5137 (0.0107)	0.7143 (0.0120)	0.7063 (0.0117)	0.7262 (**0.0114**)
Avg.Acc	0.7246	0.7245	0.7348	0.7018	0.5527	0.7321	**0.7356**	0.7306
Avg.Std	0.0106	0.0116	0.0094	0.0096	0.0074	0.0091	0.0092	**0.0090**

The best results are highlited in this table.

**Table 4:** Average accuracies of four different source domains for the transfer learning methods.

Target domain	Au-SVM	Tr-Adaboost	ELSR-TL (NN)	ELSR-TL (TSK)	ELSR-TL (Ker)
aa	0.6583(0.0165)	0.6630(0.0242)	0.7320(0.0116)	0.7332(0.0108)	**0.7352(0.0108)**
al	**1(0)**	0.9955(0.0002)	**1(0)**	**1(0)**	**1(0)**
av	0.5725(0.0180)	0.5561(0.0146)	0.5840(**0.0101**)	**0.5935**(0.0114)	0.5887(0.0113)
aw	0.7359(0.0179)	0.7389(0.0117)	**0.7939**(0.0108)	0.7907(0.0117)	0.7929(**0.0100**)
ay	0.7838(0.0184)	0.7460(0.0173)	0.8277(0.0111)	**0.8433(0.0110)**	0.8380(0.0110)
Avg.Acc	0.7501	0.7399	0.7875	**0.7921**	0.7910
Avg.Std	0.0142	0.0136	0.0087	0.0090	**0.0086**

The best results are highlited in this table.

In summary, we show that ELSR-TL-based methods can outperform other methods, especially when the number of training samples in the target domain is limited.

### 4.5 On different feature extraction

**Figure 7:**
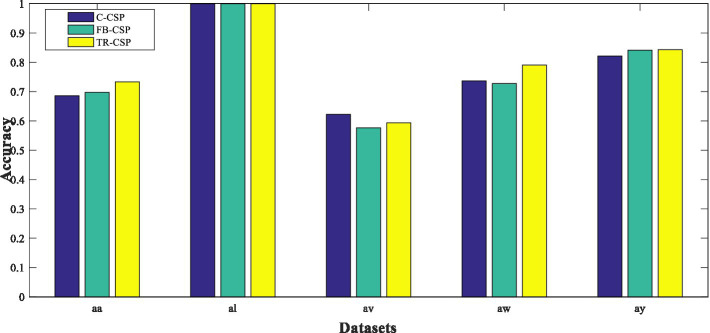
Classification results of three feature extraction methods.

In this section, we compare the effectiveness of three feature extraction methods. Figure 7 illustrates the classification results of these methods when using the same classification method. Specifically, we use ELSR-TL (TSK) as the classification method.

### 4.6 On running time

In this section, we compared the average running times of all adopted methods (without CNN) over ten trials. Table 5 lists the average time (in seconds) for each method across all datasets. It is evident that LSR has the shortest computational time. However, among all transfer learning methods, the computational time of ELSR-TL (NN) is less than that of the other two transfer learning methods. Nevertheless, the running time of the proposed ELSR-TL is still not particularly small. Therefore, determining how to accelerate the proposed method for large-scale data remains an open problem that we should explore in the future.

**Table 5:** Running time (Seconds) for all adopted methods on all datasets.

Datasets	LSR	kNN	SVM	NB	ELSR(NN)	ELSR(TSK)	ELSR(Ker)	Au-SVM	Tr-Adaboost	ELSR-TL (NN)	ELSR-TL (TSK)	ELSR-TL (Ker)
aa	0.2715	0.3014	83.9542	0.3058	84.5401	85.0113	86.7844	823.7881	325.6131	269.3713	274.3649	298.0787
al	0.2646	0.3001	83.0776	0.3022	84.5932	84.9688	85.0619	819.3875	325.2478	269.0109	271.3828	293.1613
av	0.2503	0.2953	82.5644	0.2759	82.9663	83.6533	83.9803	818.7702	310.2496	260.4783	270.5006	289.3647
aw	0.2382	0.2922	82.1311	0.2801	80.6762	81.4226	82.2949	803.2313	298.1549	252.3586	266.1136	279.5312
ay	0.2344	0.2887	79.6846	0.2794	78.4760	78.6811	79.1756	801.4153	296.9221	249.3428	258.9078	268.5438

### 4.7 Statistical analysis

A nonparametric Friedman test (Zhang Y. et al., 2023) is used to validate whether the performance differences among different algorithms are statistically significant. This test uses the rankings of different algorithms in multiple comparisons. First, we calculate the sum ranking and average ranking of the accuracy of each algorithm (without CNN), as shown in Table 6, and find the best one. We then perform *post hoc* hypothesis testing.

**Table 6:** Rankings of the 12 algorithms (Friedman test).

Algorithm	aa	al	av	aw	ay	Sum Ranking	Average Ranking
LSR	7	5.5	12	10	11	45.5	9.1
kNN	12	5.5	5	7	7	36.5	7.3
SVM	11	12	8.5	9	5	45.5	9.1
NB	5	5.5	10	12	12	44.5	8.9
ELSR(NN)	8	5.5	7	8	9	37.5	7.5
ELSR(TSK)	6	5.5	6	5	10	32.5	6.5
ELSR(Ker)	4	5.5	11	11	8	39.5	7.9
Au-SVM	10	5.5	4	6	4	29.5	5.9
Tr-Adaboost	9	11	8.5	4	6	38.5	7.7
ELSR-TL(NN)	3	5.5	3	1	3	15.5	3.1
ELSR-TL(TSK)	2	5.5	1	3	1	12.5	2.5
ELSR-TL(Ker)	1	5.5	2	2	2	12.5	2.5

The Friedman test statistics are as Equation 29:

(29) $$Q=\frac{12}{kn\left(n+1\right)}\overset{k}{\underset{i=1}{\sum }}\left(R_{i}-\frac{k{\left(n+1\right)}^{2}}{2}\right)=\frac{12}{kn\left(n+1\right)}\overset{k}{\underset{i=1}{\sum }}R_{i}^{2}-3k\left(n+1\right)$$

where $R_{i}$ is the sum ranking of each algorithm, *n* is the number of algorithms, and k is the number of datasets.

From Table 6, we have Q=26.25 , and the corresponding *p*-value is 0.005964. This suggests that the performance differences among the 12 methods are statistically significant, with ELSR-TL (TSK) performing the best. To further evaluate the performance differences between ELSR-TL (TSK) and the other 11 methods, we also conduct post hoc multiple comparison tests:

$z=\frac{\left|{\bar{R}}_{0}-{\bar{R}}_{i}\right|}{SE}$ with $SE=\sqrt{\frac{n^{*}\left(n+1\right)}{6^{*}k}}$

where *z* is subject to the standard normal distribution that will be used further to calculate the value of *P*.

Table 7 shows the *post hoc* comparison results for α=0.05 (Friedman). The null hypothesis is rejected when p≤0.00625 because p≤Holm . In summary, we conclude that there are significant performance differences between ELSR-TL (TSK) and other methods, confirming that transfer learning is effective in boosting classification accuracy.

**Table 7:** The *post-hoc* comparison for α=0.05 (Friedman).

*i*	Algorithm	*z*	*p*	Holm	Hypothesis
11	LSR	2.894291	0.0038	0.004545	Reject
10	SVM	2.894291	0.0038	0.005	Reject
9	NB	2.806586	0.005007	0.005556	Reject
8	ELSR(Ker)	2.368057	0.017882	0.00625	Reject
7	Tr-Adaboost	2.280351	0.022587	0.007143	Accept
6	ELSR(NN)	2.192645	0.028333	0.008333	Accept
5	kNN	2.104939	0.035297	0.01	Accept
4	ELSR(TSK)	1.754116	0.079411	0.0125	Accept
3	Au-SVM	1.490999	0.135962	0.016667	Accept
2	ELSR-TL(NN)	0.263117	0.79246	0.025	Accept
1	ELSR-TL(Ker)	0	1	0.05	Accept

**Figure 8:**
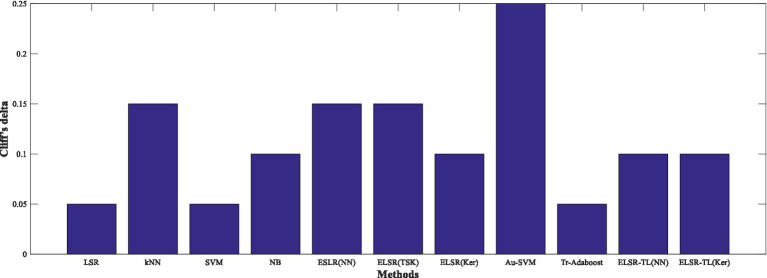
Specific differences of ELSR-TL (TSK) compared to other methods.

In addition, Figure 8 shows the specific differences between ELSR-TL (TSK) and other methods. Clearly, it is consistent with the above conclusion that there are significant performance differences between ELSR-TL (TSK) and the other methods.

### 4.8 Sensitivity analysis

**Figure 9:**
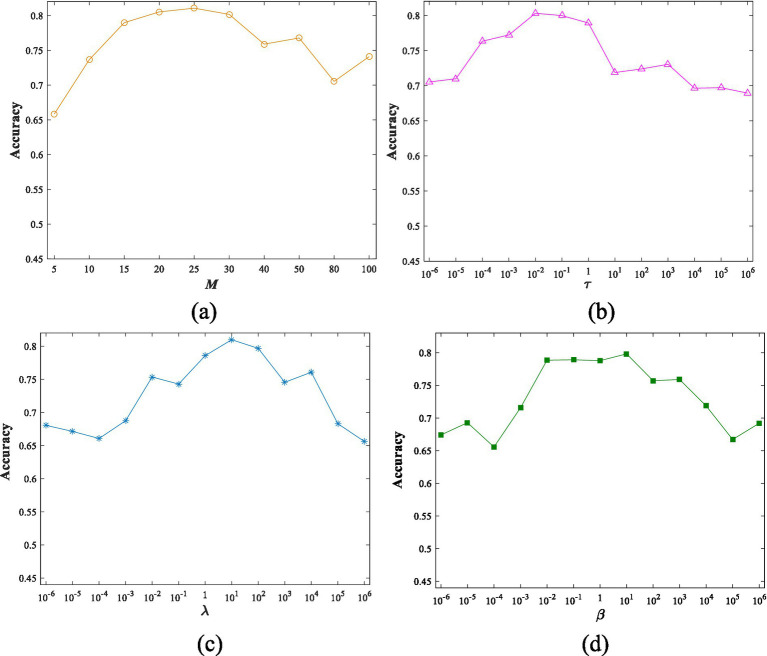
Accuracy changes with different values of four parameters: **(a)** *M*, **(b)** τ , **(c)** λ , **(d)** β .

We also conduct experiments to study the sensitivity of ELSR-TL to various parameters. Below, we use the AW dataset as an example of sensitivity analysis. Figure 9 illustrates how accuracy varies with different values of four parameters while the others remain fixed, based on the grid search detailed in section 4.2. Please note that, due to the limitations of this paper, we only use ELSR-TL (TSK) for the sensitivity analysis.

### 4.9 Limitations

Although the proposed ELSR-TL demonstrates effectiveness in these experiments, it still has some limitations. For example, there are four hyperparameters in the proposed method, and the hyperparameter optimization procedure based on grid searching and cross-validation is computationally expensive. The running time of the proposed method is significant, especially compared to traditional simple methods, making it unsuitable for real-time scenarios. The proposed ELSR-TL operates offline and cannot be used in online scenarios. The datasets used in this paper contain only five subjects on a small scale, and the effectiveness of the proposed method needs to be validated on more extensive and larger datasets in future studies. In addition, it is also worth further investigating how to provide more theoretical justifications for knowledge transfer and how to avoid negative transfer. We primarily focused on binary MI tasks in this study; therefore, exploring how to extend the proposed ELSR-TL to multi-class MI tasks, multimodal integration, and cross-dataset transfers is worth studying.

## 5 Conclusion

In this study, an extended LSR-based inductive transfer learning method was proposed to facilitate transfer learning for several classical intelligent models, including neural networks, TSK fuzzy systems, and kernel methods. We applied this method to MI-EEG signal recognition in BCIs. ELSR-TL provides three distinctive advantages: (1) It features an inductive transfer learning mechanism that allows for the transfer of useful knowledge from the source domain to enhance learning performance in the target domain when the training data in the target domain are insufficient. (2) It enhances application and generalization by extending LSR while integrating multiple classic base models such as neural networks, TSK fuzzy systems, and kernel methods. (3) It uses knowledge extracted from the source domain to train the classification model in the target domain, ensuring security for MI-EEG signal recognition. Experimental studies indicate the effectiveness of the proposed method in MI-EEG signal recognition. Although the proposed ELSR-TL demonstrates effectiveness in these experiments, there is still room for further research. For example, the hyperparameter optimization procedure based on grid searching and cross-validation is computationally expensive, so future research should focus on addressing this issue. The proposed ELSR-TL operates offline and cannot be applied in real-time scenarios. The datasets used in this study are relatively small in scale; thus, the effectiveness of the proposed method needs validation on more extensive datasets in future studies. Additionally, it is also worth further examining how to provide more theoretical justifications for knowledge transfer to avoid negative transfer. While this study primarily focuses on binary MI tasks, extending the proposed ELSR-TL to multi-class MI tasks, multimodal integration, and cross-dataset transfers is also worth studying.

## Data Availability

Publicly available datasets were analyzed in this study. This data can be found at: https://www.bbci.de/competition/iii/desc_IVa.html.
